# Active Nanofibrous Membrane Effects on Gingival Cell Inflammatory Response

**DOI:** 10.3390/ma8105376

**Published:** 2015-10-27

**Authors:** David-Nicolas Morand, Olivier Huck, Laetitia Keller, Nadia Jessel, Henri Tenenbaum, Jean-Luc Davideau

**Affiliations:** 1 INSERM (French National Institute of Health and Medical Research), UMR 1109, Osteoarticular and Dental Regenerative Nanomedicine laboratory, Faculté de Médecine, FMTS, Strasbourg Cedex F-67085, France; huck.olivier@gmail.com (O.H.); lkeller@unistra.fr (L.K.); nadia.jessel@inserm.fr (N.J.); htenen@gmail.com (H.T.); jldcabfra@wanadoo.fr (J.-L.D.); 2 Department of Periodontology, University of Strasbourg, Dental Faculty, 8 rue Sainte-Elisabeth, Strasbourg F-67000, France

**Keywords:** anti-inflammatory agents, lipopolysaccharides, fibroblasts, epithelial cells, polycaprolactone, cytokines

## Abstract

Alpha-melanocyte stimulating hormone (α-MSH) is involved in normal skin wound healing and also has anti-inflammatory properties. The association of α-MSH to polyelectrolyte layers with various supports has been shown to improve these anti-inflammatory properties. This study aimed to evaluate the effects of nanofibrous membrane functionalized with α-MSH linked to polyelectrolyte layers on gingival cell inflammatory response. Human oral epithelial cells (EC) and fibroblasts (FB) were cultured on plastic or electrospun Poly-ε-caprolactone (PCL) membranes with α-MSH covalently coupled to Poly-*L*-glutamic acid (PGA-α-MSH), for 6 to 24 h. Cells were incubated with or without *Porphyromonas gingivalis* lipopolysaccharide (*Pg*-LPS). Cell proliferation and migration were determined using AlamarBlue test and scratch assay. Expression of interleukin-6 (IL-6), tumor necrosis factor (TNF-α), and transforming growth factor-beta (TGF-β) was evaluated using RT-qPCR method. Cell cultures on plastic showed that PGA-α-MSH reduced EC and FB migration and decreased IL-6 and TGF-β expression in *Pg*-LPS stimulated EC. PGA-α-MSH functionalized PCL membranes reduced proliferation of *Pg*-LPS stimulated EC and FB. A significant decrease of IL-6, TNF-α, and TGF-β expression was also observed in *Pg*-LPS stimulated EC and FB. This study showed that the functionalization of nanofibrous PCL membranes efficiently amplified the anti-inflammatory effect of PGA-α-MSH on gingival cells.

## 1. Introduction

Periodontal wound healing involves complex interactions between different cell types (epithelial cells, fibroblasts, osteoblasts, and cementoblasts) [1] and synthesis of mediators such as growth factors and cytokines [2]. After conventional periodontal therapy, wound healing corresponds more to tissue reparation than regeneration [1]. This absence of true regeneration is considered to be mainly due to the tissue competition between the different periodontal tissues (gingiva, cementum, and alveolar bone) and the various rate of proliferation, migration and differentiation of periodontal cells during wound healing [2,3]. Furthermore, the inflammatory response could also interfere with the healing process via the modification of pro-/anti-inflammatory cytokines expression in periodontal tissues [4,5]. Indeed, major factors involved in periodontal tissue inflammation/destruction, such as interleukin-6 (IL-6), tumor necrosis factor (TNF-α) [6], and in periodontal tissue formation such as transforming growth factor-beta (TGF-β) [7] could modulate periodontal healing [8]. TNF-α and IL-6 play a central role in the inflammatory reaction, alveolar bone resorption and in connective tissue attachment loss [6]. TGF-β is a major regulator of cell replication and differentiation in periodontal wound healing [9]. All these data suggest that the control of inflammatory response could improve periodontal healing and regeneration as shown for epidermal keratinocytes and fibroblasts [9].

The anti-inflammatory properties of alpha-melanocyte stimulating hormone (α-MSH) have recently been demonstrated during cutaneous wound healing *in vivo* [10] and in dental pulp fibroblasts stimulated by *Porphyromonas gingivalis* lipopolysaccharide (*Pg*-LPS) *in vitro* [11]. α-MSH is an endogenous 13 amino acid neuropeptide derived from proteolytic cleavage of pro-opiomelanocortin hormone, which is synthesized by normal human keratinocytes and melanocytes [12]. α-MSH binds to a melanocortin receptor and was originally characterized as a regulator of pigmentation and the production of cortisol [13]. α-MSH also down-regulates pro/anti-inflammatory cytokines such as IL-6, TGF-β, and TNF-α inhibiting Nuclear Factor kappa B (NF-κB) in epidermal keratinocytes [14] and fibroblasts [15].

In order to induce periodontal tissue regeneration, many devices including or not various regenerative molecules have been used for a long time, such as membranes [1]. Initially, membranes were used as physical barriers between gingiva and underlying periodontal tissues, to allow cementum and bone forming cells to migrate onto the root surface and adequately form connective attachment [1]. These membranes have also been used more or less successfully as controlled delivery systems for growth factors. The efficiency of these membranes could be increased by adjusting biocompatibility, size, organization of the fibers and control of release time, and local levels of growth and differentiation factors [1]. Poly-ε-caprolactone (PCL) membranes are already approved for medical use by the FDA (United States Food and Drug Administration). PCL membranes are bio-resorbable and have been shown to effectively induce bone regeneration [16] and periodontal tissue regeneration [17]. PCL membranes show a better adhesion/growth of cells compared to collagen membranes, which are commonly used for the periodontal regeneration [18]. Furthermore, electrospinning procedures allow the production of nonwoven scaffolds based on PCL nanofibers of various sizes mimicking the fibrillar organization of extracellular matrix [16]. To make these electrospun nanofibrous membranes active, polyelectrolyte multilayered technologies could be used such as the coating of nanofibers with nanoreservoirs [19].

Previous studies in our laboratory have shown that α-MSH coupled to Poly-*L*-glutamic acid (PGA) has the same biological properties than native α-MSH and allows its association with various supports [11,20]. Furthermore, PGA-α-MSH associated to polyelectrolyte multilayers induces anti-inflammatory reaction of dental pulp fibroblasts quickly [11] and reduces inflammatory reaction to tracheal prosthesis in rats [20]. Therefore, the aim of this study was to investigate the short-term effects of PGA-α-MSH associated to nanofibrous PCL membranes on IL-6, TGF-β, and TNF-α expression in human oral epithelial cells and fibroblasts pre-stimulated with *Pg*-LPS.

## 2. Results α-MSH 

### 2.1. PGA-α-MSH Localization on EC and FB

**Figure 1:**
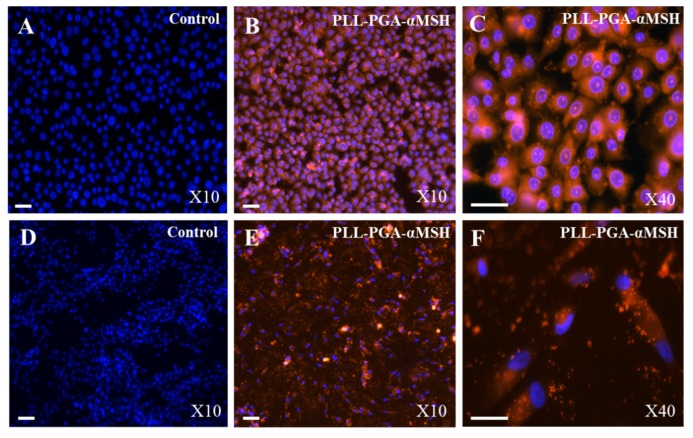
Immunolocalization of alpha-melanocyte stimulating hormone coupled to poly-*L*-glutamic and poly-*L*-lysine acid (PLL-PGA-α-MSH). Immunofluorescent analysis of human oral epithelial cells (**A**–**C**) and fibroblasts (**D**–**F**), using PLL-PGA-α-MSH conjugated to rhodamine Red after 24 h. Blue pseudocolor = DAPI (fluorescent DNA dye). All the images were imaged under the same magnification and the scale bar in all the images represents 30 μm.

Immunolocalization of PGA-α-MSH appeared homogeneously distributed in both EC and FB cell membranes. Furthermore, PGA-α-MSH did not affect the basic functions of EC and FB, such as morphology and cytoskeletal organization. Finally, the data suggested that the PGA-α-MSH was not cytotoxic for these cells (Figure 1).

### 2.2. Effect of PGA-α-MSH on Cell Proliferation/Viability and Migration

**Figure 2:**
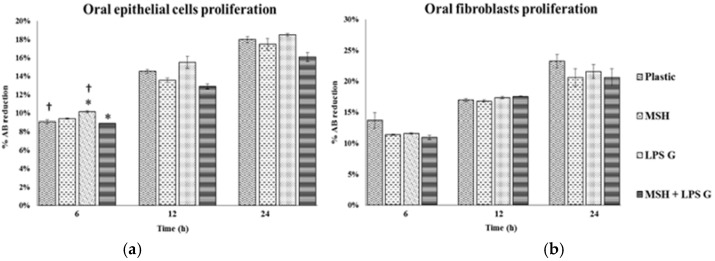
Human oral epithelial cells (**a**); and fibroblasts (**b**) proliferation. Proliferation of human oral epithelial cells and fibroblasts were analyzed after 6, 12 and 24 h on cell cultures (*n* = 4). These different conditions have been measured by using AlamarBlue test. Data are expressed as mean ± SD. †: difference between non-stimulated and stimulated cells, *p* < 0.05, *: difference between stimulated cells with or without PGA-α-MSH, *p* < 0.05.

The proliferation rates of EC and FB were investigated and compared at 6, 12 and 24 h in cell cultures. Proliferation rates of both cell types increased with time whatever cell culture conditions. *Pg*-LPS stimulation had a tendency to increase EC proliferation and to decrease FB proliferation. PGA-α-MSH decreased proliferation in *Pg*-LPS stimulated EC (Figure 2).

**Figure 3:**
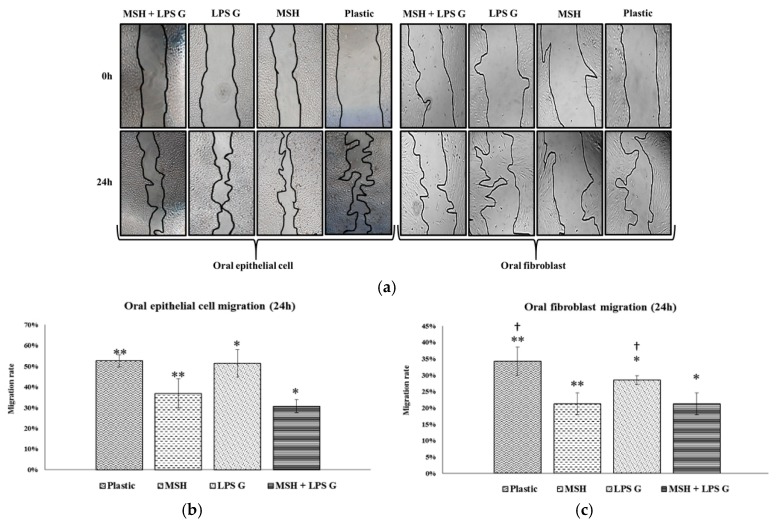
Human oral epithelial cells and fibroblasts migration. Representative images of scratch closure in the *in vitro* scratch assay (**a**). Quantification of human oral epithelial cells (**b**) and fibroblast migration (**c**) at 24 h on cell cultures (*n* = 5). Data are expressed as mean ± SD. †: difference between non-stimulated and stimulated cells, *p* < 0.05; **: difference between non stimulated cells with or without PGA-α-MSH; *: difference between stimulated cells with or without PGA-α-MSH, *p* < 0.05.

Concerning the migration rate, scratch assay showed that PGA-α-MSH decreases significantly EC and FB migration at 24 h in non-stimulated and stimulated cell cultures. *Pg*-LPS reduced FB migration (Figure 3). The profiles of cell migration and proliferation were similar at 24 h. These results showed that PGA-α-MSH at concentration of 50 µg/mL was not cytotoxic, homogenously distributed and exerted significant influence on proliferation and migration of EC and FB stimulated or not stimulated by *Pg*-LPS.

### 2.3. Effect of PGA-α-MSH on Expression Profiles of IL-6, TGF-β and TNF-α in EC and FB

**Figure 4:**
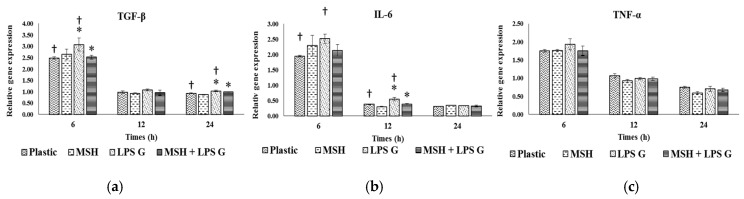
Gene expression of transforming growth factor-beta (TGF-β) (**a**); interleukin-6 (IL-6) (**b**); tumor necrosis factor (TNF-α) (**c**) in human oral epithelial cells. Relative mRNA levels were analyzed by real-time quantitative RT-PCR for IL-6, TGF-β and TNF-α in EC after 6, 12 and 24 h on cell cultures (n = 4). Data are expressed as mean ± SD. †: difference between non-stimulated and stimulated cells, *p* < 0.05; *: difference between stimulated cells with or without PGA-α-MSH, *p* < 0.05.

**Figure 5:**
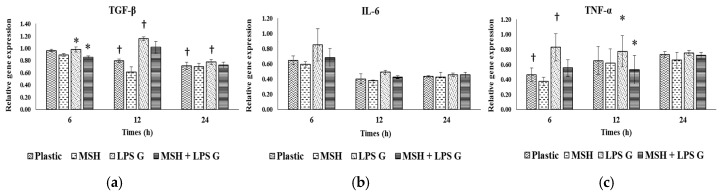
Gene expression of TGF-β (**a**); IL-6 (**b**); and TNF-α (**c**) in human oral fibroblasts. Relative mRNA levels were analyzed by real-time quantitative RT-PCR for IL-6, TGF-β and TNF-α in FB after 6, 12 and 24 h on cell cultures (*n* = 4). Data are expressed as mean ± SD. †: difference between non-stimulated and stimulated cells, *p* < 0.05; *: difference between stimulated cells with or without PGA-α-MSH, *p* < 0.05.

To evaluate the effect of PGA-α-MSH on pro-inflammatory factors, relative gene expression for IL-6, TGF-β and TNF-α of EC (Figure 4) and FB (Figure 5) were assessed at 6, 12 and 24 h in EC and FB cultures. The mean expression level of the investigated genes was generally higher in EC than in FB, especially at 6 h. The mRNA levels did not vary significantly (TGF-β, TNF-α) or slightly decreased (IL-6) with time in FB, while a more marked reduction with time was observed in EC, especially for IL-6.

#### 2.3.1. Effects of *Pg*-LPS Stimulation

Stimulation with *Pg*-LPS significantly increased gene expression in EC at different time-points, for IL-6 and TGF-β while no effect was observed for TNF-α. In FB, this effect was observed at 6 h for TNF-α, at 12 h for TGF-β while no significant effect was observed for IL-6.

#### 2.3.2. Effects of PGA-α-MSH

PGA-α-MSH had no effect on mRNA expression in non-stimulated EC and FB compared to controls. In EC *Pg*-LPS stimulated cells, PGA-α-MSH significantly decreased IL-6 and TGF-β expression at 6 and 12 h. In FB, this effect was observed for TNF-α and TGF-β mRNAs. These results showed the anti-inflammatory properties of PGA-α-MSH on stimulated gingival cells. This effect appeared cell and time dependent.

### 2.4. PGA-α-MSH Localization and Morphology Analysis of EC, FB on PCL Membranes

**Figure 6:**
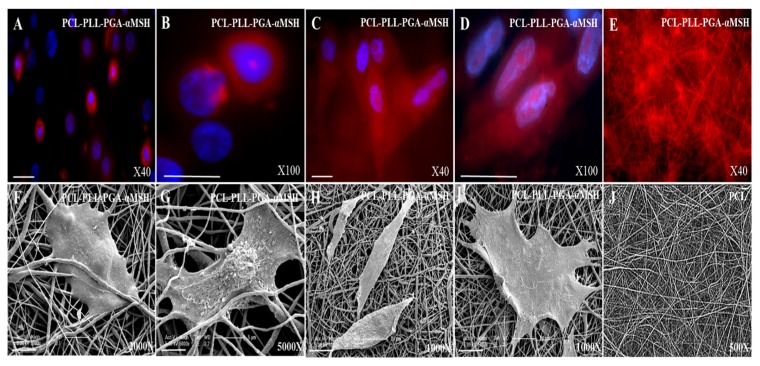
Immunofluorescent and Scanning Electron Micrograph (SEM). Analysis of human oral epithelial cells (**A**,**B**,**F**,**G**), and fibroblasts (**C**,**D**,**H**,**I**) on poly-ε-caprolactone (PCL) membrane functionalized by PLL-PGA-α-MSH after 24 h. PCL membranes is used as the control (**E**,**J**). PLL-PGA-α-MSH is conjugated to rhodamine Red. Blue pseudocolor = DAPI (fluorescent DNA dye). All the images were imaged under the same magnification and the scale bar in immunofluorescent images represents 30 μm and 5 µm (5000×), 10 µm (2000×), 20 µm (1000×) for the SEM images.

Immunofluorescent analysis showed that PGA-α-MSH appeared homogeneously distributed in both EC and FB cell membranes, as well as on the PCL membranes. Furthermore, SEM analysis showed that PGA-α-MSH does not influence morphology or adherence of EC and FB (Figure 6). Finally, the data showed a great targeted delivery of PGA-α-MSH into the cells cytosol and a great biocompatibility of PCL membranes functionalized by PGA-α-MSH.

### 2.5. Effect of PCL Membranes Functionalized with PGA-α-MSH on Cell Proliferation

**Figure 7:**
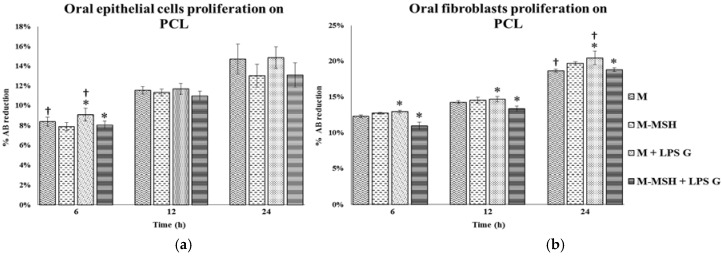
Human oral epithelial cells (**a**); and fibroblasts (**b**) proliferation with PCL membranes. Proliferation of human oral epithelial cells and fibroblasts were analyzed after 6, 12 and 24 h on PCL cultures (*n* = 4). These different conditions have been measured by using AlamarBlue test. Data are expressed as mean ± SD; †: difference between non-stimulated and stimulated cells, *p* < 0.05; *: difference between stimulated cells with or without PGA-α-MSH, *p* < 0.05.

As shown in cell cultures without PCL, the proliferation rates of both cell types increased with time whatever cell culture conditions. *Pg*-LPS stimulation increased cell proliferation of both EC and FB. PCL membranes functionalized with PGA-α-MSH decreased proliferation in *Pg*-LPS stimulated FB (Figure 7). These results showed that functionalized PCL membranes were biocompatible and exerted little influence on proliferation of non-stimulated and *Pg*-LPS stimulated FB.

### 2.6. Effect of PCL Membranes Functionalized with PGA-α-MSH on Expression profile of IL-6, TGF-β and TNF-α in EC and in FB

**Figure 8:**
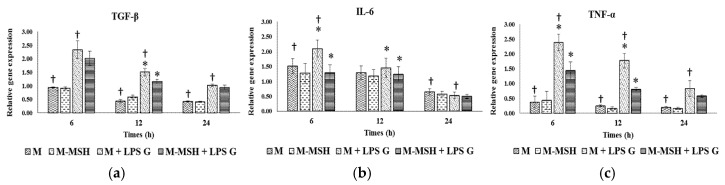
Gene expression of TGF-β (**a**); IL-6 (**b**); and TNF-α (**c**) in human oral epithelial cells with PCL membranes. Relative mRNA levels were analyzed by real-time quantitative RT-PCR for IL-6, TGF-β and TNF-α in EC after 6, 12 and 24 h on PCL cultures (*n* = 4). Data are expressed as mean ± SD. †: difference between non-stimulated and stimulated cells, *p* < 0.05; *: difference between stimulated cells with or without PGA-α-MSH, *p* < 0.05.

**Figure 9:**
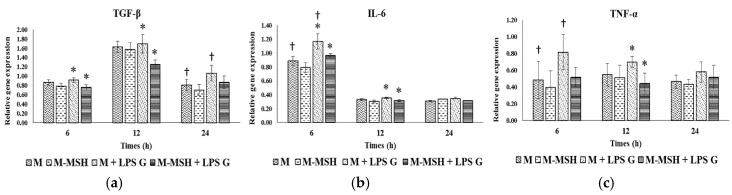
Gene expression of TGF-β (**a**); IL-6 (**b**); and TNF-α (**c**) in human oral fibroblasts with PCL membranes. Relative mRNA levels were analyzed by real-time quantitative RT-PCR for IL-6, TGF-β and TNF-α in EC after 6, 12 and 24 h on PCL cultures (*n* = 4). Data are expressed as mean ± SD. †: difference between non-stimulated and stimulated cells, *p* < 0.05; *: difference between stimulated cells with or without PGA-α-MSH, *p* < 0.05.

To evaluate the effect of PCL membranes functionalized with PGA-α-MSH on pro-inflammatory factors, relative gene expression for IL-6, TGF-β and TNF-α of EC (Figure 8) and FB (Figure 9) were assessed at 6, 12 and 24 h in PCL cultures. The level of TGF-β and TNF-α expression of FB and EC was similar while a higher expression of IL-6 was observed in EC. Considering expression changes with time, mRNA levels progressively decreased with time (IL-6) or remained stable (TGF-β, TNF-α) whatever cell types and culture conditions. However a peak of TGF-β expression was observed in FB at 12 h.

#### 2.6.1. Effects of *Pg*-LPS Stimulation

Stimulation with *Pg*-LPS significantly increased IL-6, TNF-α, and also TGF-β expression in EC at different time-points, especially for TNF-α. In FB, this effect was only observed at 6 h for IL-6 and TNF-α, and at 24 h for TGF-β and was less pronounced. Indeed, at 6 h, *Pg*-LPS induced a six-fold increase of TNF-α in EC *versus* a 2-fold increase in FB.

#### 2.6.2. Effects of PGA-α-MSH

As shown for cell cultures without PCL, PCL membranes functionalized with PGA-α-MSH had no effect on mRNA expression in non-stimulated EC and FB compared to controls. In *Pg*-LPS stimulated cells, functionalized PCL membranes with PGA-α-MSH significantly decreased studied gene expression at 6 and 12 h but not at 24 h. In FB, PCL membranes functionalized with PGA-α-MSH returned from mRNA levels of stimulated cells to mRNA levels of non-stimulated cells. However, in EC, the levels of TNF-α and TGF-β mRNAs remained higher in stimulated than in non-stimulated cultured cells on PCL membranes functionalized with PGA-α-MSH.

These results showed the anti-inflammatory properties of PCL membranes functionalized with PGA-α-MSH in stimulated gingival cells. This effect was time dependent and appeared more efficient in FB than in EC.

### 2.7. Specific Effect of PCL Membranes on EC and FB Responses to PGA-α-MSH

The proliferation rate was higher in cell culture on plastic than on PCL at 12 and 24 h, especially for EC. Interestingly, the effect of *Pg*-LPS stimulation on FB proliferation in cell cultures on plastic was inverted in PCL cultures.

The basal levels of TGF-β, TNF-α mRNAs were significantly lower for PCL than for cell cultures on plastic while mRNAs levels were similar in FB. The variations with time of gene expression between cell cultures on plastic and PCL membranes were globally similar with a maximum of gene expression at 6 h. However, a maximum of TGF-β expression was specifically observed in FB cultures with PCL membranes at 12 h. These data showed that PCL membranes had *per se* an anti-inflammatory effect, specifically for EC. The effects of PGA-α-MSH on *Pg*-LPS stimulated cell gene expression were similar in cell cultures with or without PCL membranes except for TNF-α in EC and IL-6 in FB. The association of PGA-α-MSH PCL membranes seemed to differentially potentiate its anti-inflammatory effect depending on cell and cytokine types.

## 3. Discussion

In the present study, the effect of α-MSH on gingival cell inflammatory response was investigated for the first time. Alpha-MSH and/or PGA-α-MSH have been previously shown to decrease inflammation response in pulp fibroblasts and skin epithelial cells [11,21]. Our data showed that PGA-α-MSH decreased the migration of EC and FB pre-stimulated by *Pg*-LPS. Previous studies have shown that α-MSH at nanomolar doses inhibits activation of NF-κB and consequently suppresses the expression of intercellular adhesion molecule-1 (ICAM-1) and integrin induced by pro-inflammatory stimuli such as LPS [22]. This certainly raises the possibility that the processes of proliferation, migration may be inhibited by NF-κB. An inhibition of melanoma cell migration by α-MSH has been also observed in another inflammatory context [23]. Therefore, cell modulation of proliferation and migration by α-MSH may play a role in the anti-*s*oft tissues invasion and anti-fibrotic effects into the wound site [10].

*Pg*-LPS is a strong inducer of pro-inflammatory responses in gingival epithelial and fibroblastic cells [24,25,26]. In EC and FB cultures, *Pg*-LPS pre-treatment increased IL-6, TNF-α, and TGF-β, as previously observed in gingival fibroblasts and epithelial cells [25,26,27]. However, the increase of TGF-β and TNF-α after *Pg*-LPS stimulation was more pronounced in EC than in FB. These results were comparable to those observed in keratinocytes and fibroblasts in skin substitutes [28]. This difference in expression could be due in part to the type of toll-like receptor (TLR) activated by *Pg*-LPS [24]. Indeed, studies showed that *Pg*-LPS used TLR2 in EC [24] and TLR4 in FB [29] for initiating an inflammatory response. Depending on the activated signaling pathway, expression profiles of pro-inflammatory cytokines will be different [30]. Our results showed that PGA-α-MSH down-regulated IL-6, TGF-β, and TNF-α expression induced by *Pg-*LPS stimulation in EC and FB cultures. Similar results have been demonstrated *in vitro* studies with human bronchial epithelial cells, pre-stimulated with *Pg*-LPS [21] or on fibroblasts, pre-stimulated with *E. Coli*-LPS [11] and with keratinocytes activated by *S. aureus* [31]. These data showed the anti-inflammatory properties PGA-α-MSH in gingival cells and that this effect was time dependent and appeared more efficient in FB than in EC.

Previous studies have shown that α-MSH and/or PGA-α-MSH anti-inflammatory effect could be improved by its association to various supports/scaffolds, as observed for the association of PGA-α-MSH with polyelectrolyte multilayers in dental pulp fibroblasts [11]. PCL membranes have been previously proposed/used to control periodontal ligament and bone healing [17] and their biocompatibility has been already demonstrated in other cell types such as osteoblasts *in vitro* [16] and *in vivo* [17]. In the present study, the proliferation rate and cell morphology of EC and FB cultured on PCL membranes showed that PCL membranes were biocompatible for gingival cells. Interestingly, the use of PCL membranes delayed the increase of cell proliferation rate with time as shown for other membranes [32], and this effect appeared more pronounced in EC than in FB. This difference in proliferation between EC and FB may be related to the surface topography and chemistry (wettability, softness and stiffness, and roughness), microstructure (porosity, pore size, pore shape, interconnectivity, and specific surface area), and mechanical properties of the cultures [33]. Indeed, L929 fibroblastic cells preferred rough and porous structures with higher hydrophobicity in contrast to MDBK epithelial cells, which preferred smooth surface or low level of roughness [34]. The nanofibrous structure of the PCL membranes used here may explain these data. Furthermore, the proliferation decrease of *Pg*-LPS stimulated FB with PCL membranes functionalized with PGA-α-MSH suggested that these membranes may prevent or delay gingival cell migration in an inflammatory context as observed here in EC and FB cultures with PGA-α-MSH.

Regarding the inflammatory properties of PCL membranes functionalized with PGA-α-MSH, our results showed that the gene down-regulation induced by PGA-α-MSH in gingival cell cultures on plastic persisted and in some case was amplified using functionalized PCL membranes with the same dose of PGA-α-MSH. The association of molecules and drugs with various supports has been shown to improve their biological action [16,20] but not systematically. For instance, the anti-invasive properties of risedronate embedded in various polyelectrolyte multilayers were reduced compared to the molecule in solution [35]. In the present study, the use of PCL membranes functionalized with PGA-α-MSH generally amplified and/or extended over time the anti-inflammatory effect of PGA-α-MSH. However, the intensity of induced down-regulation varied depending on cell type. In EC, PGA-α-MSH partly counterbalanced the increase of TGF-β and TNF-α induced by *Pg*-LPS while in FB, PGA-α-MSH could depress TGF-β expression under basal level. These results could not be explained by the number of melanocortin receptors in EC and FB while this receptor appeared more expressed in keratinocytes [12], but could be due to the over stimulation by *Pg*-LPS of TGF-β and TNF-α in EC. The choice of inflammation inducer has been also suggested to influence the effect of PGA-α-MSH [11].

Interestingly, nanofibrous PCL membranes use here modified *per se* quantitatively and qualitatively inflammatory responses. Indeed, PCL membranes markedly reduced the expression of the investigated genes compared to gingival cell cultures on plastic, especially for EC expression of TGF-β and TNF-α. In FB, no such quantitative effect of PCL membranes on gene expression was observed but TGF-β expression was delayed with a peak of expression at 12 h. The modulation of gene expression profile has been previously demonstrated for TNF-α, IL-8 and IL-1β in human fibroblasts, chondrocytes on polylactic acid (PLA) scaffolds and two-dimensional polystyrene plates [36,37]. These variations in mRNAs expressions may be related to the effect of membrane architecture on cell inflammatory response [33].

## 4. Material and Methods

### 4.1. Electrospinning

Poly (ε-caprolactone) (Perstorp, Malmö, Sweden) was dissolved in a mixture of dichloromethane/dimethylformamide (DCM/DMF 50/50 *v*/*v*) at 15% wt/*v* and was stirred overnight before use. A standard electrospinning set-up apparatus (EC-DIG, IME Technologies, Eindhoven, the Netherlands) was used to fabricate the PCL nanofibrous membranes. The PCL solution was poured into a 5 mL syringe and ejected through a 21G needle of 0.5 mm outer diameter at a flow rate of 1.2 mL·h^−1^ via a programmable pump (ProSense, München, Germany). During the electrospinning process, a voltage of +15 kV was applied on the needle. The electrospun jet was then collected in an aluminium foils (20 × 20 cm^2^) at a distance of distance from the needle of 17 cm.

### 4.2. PCL Membranes Functionalization with PGA-α-MSH

PCL membranes were synthesized by electrospinning as previously described [16]. PCL membranes were treated with Poly-*L*-lysine hydrobromide (PLL) (Sigma, St-Quentin, France) (100 µg/mL) and functionalized at the concentration of 100 µg/mL [11] with α-MSH peptide (HS-CH2CH2-Ser-Tyr-Ser-Nle-Glu-His-D-Phe-Arg-Trp-Gly-Lys-Pro-Val-COOH) (Neosystem, Strasbourg, France) covalently coupled to Poly-*L*-glutamic acid (PGA) (Sigma, St-Quentin, France), which leaves accessible the anti-inflammatory C-terminal sequence Lys11-Pro12-Val13 [1,20]. All membranes were sterilized by 30 min exposure to UV light (254 nm, 30 W, distance 20 cm).

### 4.3. Cell Cultures

TERT-2 OKF-6 (BWH Cell Culture and Microscopy Core, Boston, MA, USA) human oral epithelial cells (EC) were cultivated in Keratinocyte-SFM medium (Life Technologies, Saint-Aubin, France) supplemented with growth supplementation mix and antibiotics (10 U/mL penicillin and 100 µg/mL streptomycin) (Lonza, Levallois-Perret, France). Human oral fibroblasts (FB) were isolated from gingival biopsy (French Ministry of Research, Bioethic department authorization DC-2014-2220) and cultivated in RPMI 1640 medium supplemented with 10% fetal bovine serum (Life Technologies), 2 mM glutamine, 250 U/mL fungizone and 10 U/mL antibiotics (10 U/mL penicillin and 100 µg/mL streptomycin) [38]. Cells were cultivated at 37° in a humidified atmosphere with 5% CO_2_. For each experiment, 5 × 10^4^ cells were seeded in each well of a 48-wells plate and incubated with or without *Pg*-LPS (1 µg/mL) (Invivogen, San Diego, CA, USA) for 6 to 24 h.

### 4.4. Cell Proliferation

Cell proliferation was determined using colorimetric AlamarBlue test (Life Technologies). After 6 h, 12 h and 24 h, 200 µL of incubation media was transferred to 96-well plates and measured at 590 and 630 nm in order to determine the percentage of AlamarBlue reduction.

### 4.5. Wound Closure Assay

Cell migration was assessed by wound-healing “scratch” assay. Cells were seeded in 48 well plates at 2.5 × 10^4^ cells/mL in their respective medium and grown until confluence. Cells were washed with PBS. In each well, a scratch was made with the tip of a sterile pipet point (200 µL). Cells were washed with PBS in order to remove cells debris. In each well, 500 µL of medium containing 50 µg/mL PGA-α-MSH or only medium was added. The scratch was captured immediately and after 24 h with an optical microscope (Nikon Eclipse TS100) and the area of the scratch was calculated with Photoshop CS4. Closure percentage of the scratch was calculated as ((surface of the scratch at time 0 h and surface of the scratch at time 24 h)/(surface of the scratch at time 0 h × 100)).

### 4.6. Immunofluorescence

The homogeneous localization of PGA-α-MSH conjugated to rhodamine Red after 24 h was determined using immunofluorescence microscopy. Gingival cells were cultured in presence of PGA-α-MSH conjugated to rhodamine Red on PCL membranes and in solution. Gingival cells were fixed with 4% paraformaldehyde for 1 h and incubated for 5 min with 200 nM DAPI (Sigma, St-Quentin, France) for nuclear staining. The samples were observed under an epifluorescence microscope (LEICA DM 4000 B, Wetzlar, Germany).

### 4.7. Scanning Electron Microscopy Observation

For cell morphological study, after fixation with paraformaldehyde at 5% in PBS for 30 min at 4 °C, and osmium tetroxide at 1% in PBS for 1 h at room temperature, the samples were dehydrated. Then the scaffolds were palladium-coated (Bal-Tec SCD 005, Jean-Luc Vonesch, IGBMC, Strasbourg, France) and observed with a scanning electron microscope in conventional mode (FEG Sirion XL, FEI, Jean-Luc Vonesch, IGBMC, Strasbourg, France).

### 4.8. RNA Isolation and Reverse Transcription

After cell lysis, total RNA was extracted using the High Pure RNA isolation kit (Roche Applied Science, Meylan, France) according to the manufacturer’s instructions. The extracted total RNA concentration was quantified using NanoDrop 1000 (Fischer Scientific, Illkirch, France). Reverse transcription was performed with the iScript Reverse Transcription Supermix (Bio-Rad Laboratories, Hercules, CA, USA) according to the manufacturer’s instructions.

### 4.9. Real-Time Quantitative RT-PCR Analysis (RT-qPCR)

To quantify RNA expression, RT-qPCR was performed on the cDNA samples. PCR amplification and analysis were achieved using the CFX Connect™ Real-Time PCR Detection System (Bio-rad, Miltry-Mory, France). Amplification reactions have been performed using iTaq Universal SYBR Green Supermix (Bio-rad, Miltry-Mory, France). Beta-actin was used as endogenous RNA control (housekeeping gene) in the samples. Primers sequences related to TNF-α, IL-6 and TGF-β are detailed in Table 1 and were synthesized by Life technologies. The specificity of the reaction was controlled using melting curves analysis. The expression level was calculated using the comparative Ct method (2^−ΔΔCt^) after normalization to the housekeeping gene. All RT-qPCR assays were performed in triplicate and results are represented by the mean values.

**Table 1:** List of primers used in this study.

Gene Product	Primer Name	Primer Sequence
TNF-α	TNF-α-FW	CCTGCCCCAATCCCTTTATT
	TNF-α-RW	CCCTAAGCCCCCAATTCTCT
IL-6	IL-6-FW	GCCTCAGATCTCCAGTCC
	IL-6-RW	GCCTCAGATCTCCAGTCC
TGF-β	TGF-β-FW	CCCAGCATCTGCAAAGCTC
	TGF-β-RW	GTCAATGTACAGCTGCCGCA
β-actin	β-actin-FW	GATGAGATTGGCATGGCTTT
	β-actin-RW	CACCTTCACCGTTCCAGTTT

### 4.10. Statistical Analysis

All experiments were repeated at least three times and statistical analysis was performed using ANOVA mixed model and Fisher test (LSD) for pair wise comparisons (XLSTAT, Addinsoft France, Paris, France). A probability *p* value <0.05 was considered significant.

## 5. Conclusions

This study showed for the first time the biocompatibility and the anti-inflammatory effect of PCL membranes functionalized with PGA-α-MSH for human oral epithelial cells and fibroblasts. The association of α-MSH to PCL membranes efficiently modulated proliferation and inflammatory responses of gingival cells compared to α-MSH alone. To our knowledge, few studies have attempted to integrate inflammatory modulation into tissue engineering strategies. Our work suggested that the regenerative therapeutic strategy associating membranes and growth factors could be extended to the use of anti-inflammatory drugs.

## References

[1] Kim J.H., Park C.H., Perez R.A., Lee H.Y., Jang J.H., Lee H.H., Wall I.B., Shi S., Kim H.W. (2014). Advanced Biomatrix Designs for Regenerative Therapy of Periodontal Tissues. J. Dent. Res..

[2] Polimeni G., Xiropaidis A.V., Wikesjö U.M.E. (2006). Biology and principles of periodontal wound healing/regeneration. Periodontol. 2000.

[3] Ramseier C.A., Rasperini G., Batia S., Giannobile W.V. (2012). Advanced reconstructive technologies for periodontal tissue repair. Periodontol. 2000.

[4] Giannobile W.V. (2008). Host-response therapeutics for periodontal diseases. J. Periodontol..

[5] Thomas M.V., Puleo D.A. (2011). Infection, inflammation, and bone regeneration: a paradoxical relationship. J. Dent. Res..

[6] Yucel-Lindberg T., Båge T. (2013). Inflammatory mediators in the pathogenesis of periodontitis. Expert Rev. Mol. Med..

[7] Chen F.-M., Zhang J., Zhang M., An Y., Chen F., Wu Z.-F. (2010). A review on endogenous regenerative technology in periodontal regenerative medicine. Biomaterials.

[8] Zhang X., Kohli M., Zhou Q., Graves D.T., Amar S. (2004). Short- and Long-Term Effects of IL-1 and TNF Antagonists on Periodontal Wound Healing. J. Immunol..

[9] Werner S., Krieg T., Smola H. (2007). Keratinocyte-fibroblast interactions in wound healing. J. Investig. Dermatol..

[10] De Souza K.S., Cantaruti T.A., Azevedo G.M., Galdino D.A., Rodrigues C.M., Costa R.A., Vaz N.M., Carvalho C.R. (2015). Improved cutaneous wound healing after intraperitoneal injection of alpha-melanocyte-stimulating hormone. Exp. Dermatol..

[11] Fioretti F., Mendoza-Palomares C., Helms M., Alam D.A., Richert L., Arntz Y., Rinckenbach S., Garnier F., Haïkel Y., Gangloff S.C. (2010). Nanostructured assemblies for dental application. ACS Nano.

[12] Muffley L.A., Zhu K.Q., Engrav L.H., Gibran N.S., Hocking A.M. (2011). Spatial and temporal localization of the melanocortin 1 receptor and its ligand α-melanocyte-stimulating hormone during cutaneous wound repair. J. Histochem. Cytochem..

[13] Böhm M., Luger T.A., Tobin D.J., García-Borrón J.C. (2006). Melanocortin Receptor Ligands: New Horizons for Skin Biology and Clinical Dermatology. J. Investig. Dermatol..

[14] Haycock J.W., Rowe S.J., Cartledge S., Wyatt A., Ghanem G., Morandini R., Rennie I.G., MacNeil S. (2000). α-Melanocyte-stimulating Hormone Reduces Impact of Proinflammatory Cytokine and Peroxide-generated Oxidative Stress on Keratinocyte and Melanoma Cell Lines. J. Biol. Chem..

[15] Hill R.P., MacNeil S., Haycock J.W. (2006). Melanocyte stimulating hormone peptides inhibit TNF-α signaling in human dermal fibroblast cells. Peptides.

[16] Ferrand A., Eap S., Richert L., Lemoine S., Kalaskar D., Demoustier-Champagne S., Atmani H., Mély Y., Fioretti F., Schlatter G. (2014). Osteogenetic Properties of Electrospun Nanofibrous PCL Scaffolds Equipped With Chitosan-Based Nanoreservoirs of Growth Factors. Macromol. Biosci..

[17] Vaquette C., Fan W., Xiao Y., Hamlet S., Hutmacher D.W., Ivanovski S. (2012). A biphasic scaffold design combined with cell sheet technology for simultaneous regeneration of alveolar bone/periodontal ligament complex. Biomaterials.

[18] Bashutski J.D., Wang H.-L. (2009). Periodontal and endodontic regeneration. J. Endod..

[19] Mendoza-Palomares C., Ferrand A., Facca S., Fioretti F., Ladam G., Kuchler-Bopp S., Regnier T., Mainard D., Benkirane-Jessel N. (2012). Smart hybrid materials equipped by nanoreservoirs of therapeutics. ACS Nano.

[20] Schultz P., Vautier D., Richert L., Jessel N., Haikel Y., Schaaf P., Voegel J.-C., Ogier J., Debry C. (2005). Polyelectrolyte multilayers functionalized by a synthetic analogue of an anti-inflammatory peptide, alpha-MSH, for coating a tracheal prosthesis. Biomaterials.

[21] Moustafa M., Szabo M., Ghanem G.E., Morandini R., Kemp E.H., MacNeil S., Haycock J.W. (2002). Inhibition of Tumor Necrosis Factor-α Stimulated NFκB/p65 in Human Keratinocytes by α-Melanocyte Stimulating Hormone and Adrenocorticotropic Hormone Peptides. J. Investig. Dermatol..

[22] Eves P., Haycock J., Layton C., Wagner M., Kemp H., Szabo M., Morandini R., Ghanem G., García-Borrón J.C., Jiménez-Cervantes C., Mac Neil S. (2003). Anti-inflammatory and anti-invasive effects of α-melanocyte-stimulating hormone in human melanoma cells. Br. J. Cancer.

[23] Eves P.C., MacNeil S., Haycock J.W. (2006). α-Melanocyte stimulating hormone, inflammation and human melanoma. Peptides.

[24] Kocgozlu L., Elkaim R., Tenenbaum H., Werner S. (2009). Variable cell responses to P. gingivalis lipopolysaccharide. J. Dent. Res..

[25] Ara T., Kurata K., Hirai K., Uchihashi T., Uematsu T., Imamura Y., Furusawa K., Kurihara S., Wang P.-L. (2009). Human gingival fibroblasts are critical in sustaining inflammation in periodontal disease. J. Periodontal Res..

[26] Herath T.D.K., Wang Y., Seneviratne C.J., Lu Q., Darveau R.P., Wang C.-Y., Jin L. (2011). Porphyromonas gingivalis lipopolysaccharide lipid A heterogeneity differentially modulates the expression of IL-6 and IL-8 in human gingival fibroblasts. J. Clin. Periodontol..

[27] Sugiyama A., Uehara A., Iki K., Matsushita K., Nakamura R., Ogawa T., Sugawara S., Takada H. (2002). Activation of human gingival epithelial cells by cell-surface components of black-pigmented bacteria: augmentation of production of interleukin-8, granulocyte colony-stimulating factor and granulocyte-macrophage colony-stimulating factor and expression of intercellular adhesion molecule 1. J. Med. Microbiol..

[28] Spiekstra S.W., Breetveld M., Rustemeyer T., Scheper R.J., Gibbs S. (2007). Wound-healing factors secreted by epidermal keratinocytes and dermal fibroblasts in skin substitutes. Wound Repair Regen..

[29] Wang J., Hori K., Ding J., Huang Y., Kwan P., Ladak A., Tredget E.E. (2011). Toll-like receptors expressed by dermal fibroblasts contribute to hypertrophic scarring. J. Cell. Physiol..

[30] Kawai T., Akira S. (2010). The role of pattern-recognition receptors in innate immunity: update on Toll-like receptors. Nat. Immunol..

[31] Donnarumma G., Paoletti I., Buommino E., Antonietta Tufano M., Baroni A. (2004). α-MSH reduces the internalization of Staphylococcus aureus and down-regulates HSP 70, integrins and cytokine expression in human keratinocyte cell lines. Exp. Dermatol..

[32] Kasaj A., Reichert C., Götz H., Röhrig B., Smeets R., Willershausen B. (2008). *In vitro* evaluation of various bioabsorbable and nonresorbable barrier membranes for guided tissue regeneration. Head Face Med..

[33] Chang H.-I., Wang Y., Eberli D. (2011). Cell Responses to Surface and Architecture of Tissue Engineering Scaffolds. Regenerative Medicine and Tissue Engineering—Cells and Biomaterials.

[34] Gümüşderelioğlu M., Betül Kaya F., Beşkardeş I.G. (2011). Comparison of epithelial and fibroblastic cell behavior on nano/micro-topographic PCL membranes produced by crystallinity control. J. Colloid Interface Sci..

[35] Daubiné F., Cortial D., Ladam G., Atmani H., Haïkel Y., Voegel J.-C., Clézardin P., Benkirane-Jessel N. (2009). Nanostructured polyelectrolyte multilayer drug delivery systems for bone metastasis prevention. Biomaterials.

[36] Klapperich C.M., Bertozzi C.R. (2004). Global gene expression of cells attached to a tissue engineering scaffold. Biomaterials.

[37] Kwon H., Sun L., Cairns D.M., Rainbow R.S., Preda R.C., Kaplan D.L., Zeng L. (2013). The influence of scaffold material on chondrocytes in inflammatory conditions. Acta Biomater..

[38] Krisanaprakornkit S., Kimball J.R., Weinberg A., Darveau R.P., Bainbridge B.W., Dale B.A. (2000). Inducible Expression of Human?-Defensin 2 by Fusobacterium nucleatum in Oral Epithelial Cells: Multiple Signaling Pathways and Role of Commensal Bacteria in Innate Immunity and the Epithelial Barrier. Infect. Immun..

